# The relationship between accelerometer-based physical activity, sedentary behavior, and seven common geriatric syndromes: a two-sample Mendelian randomization study

**DOI:** 10.3389/fpubh.2024.1406303

**Published:** 2024-08-05

**Authors:** Jiping Chen, Yanyu Lu, JiaWei Yao, Xianliang Zhang, Yang Pan

**Affiliations:** ^1^School of Physical Education, Shandong University, Jinan, China; ^2^Department of Physical Education Teaching and Research, Guangdong Dance and Drama College, Foshan, China

**Keywords:** accelerometer-based, physical activity, sedentary behavior, geriatric syndromes, mendelian randomization

## Abstract

**Introduction:**

To investigate the causal associations between accelerometer-based physical activity (PA), sedentary behavior (SB), and seven common geriatric syndromes (GSs) (frailty, falls, delirium, urinary incontinence, dysphagia, hearing loss, and visual impairment) by Mendelian randomization (MR) analysis.

**Methods:**

Instrumental variables from a genome-wide association study were used for MR analysis. The exposure factors were three PA phenotypes (average acceleration, overall activity, and moderate-intensity activity) and one SB phenotype (SB). The outcome variables were seven common GSs. The inverse variance weighted (IVW) method was utilized for the primary MR analysis. Additionally, sensitivity, pleiotropy, and heterogeneity analyses were subsequently conducted to assess the robustness of the present study’s findings.

**Results:**

According to the primary MR results obtained using the IVW method, genetically predicted PA (average acceleration) decreased the risk of two GSs (frailty, *p* = 0.01; dysphagia, *p* = 0.03). Similarly, overall activity decreased the risk of two GSs (frailty, *p* = 0.01; delirium, *p* = 0.03), and moderate-intensity activity reduced the risk of three GSs (urinary incontinence, *p* = 0.04; hearing loss, *p* = 0.02; visual impairment, *p* = 0.01). Furthermore, SB was causally correlated with a greater risk for three GSs (frailty, *p* = 0.03; fall, *p* = 0.01; dysphagia, *p* = 0.04).

**Conclusion:**

This study provided evidence that accelerometer-based PA may be causally associated with a lower risk of GSs, while SB may increase the risk of GSs.

## 1 Introduction

The term geriatric syndromes (GSs) refers to a group of medical conditions in the older adult, such as frailty, falls, delirium, and incontinence, associated with age increase (1, 2). Due to the complex pathogenesis involving dysregulated metabolism, immune system decline, and musculoskeletal dysfunction, the incidence of GSs has shown an upward trend consistently in recent years (3). According to recent epidemiological advancements, GSs affect approximately 10–33% of adults aged 65 and above worldwide, with more than 40% experiencing two or more symptoms simultaneously (4, 5). These conditions lead to long-term disability, emotional distress, and social isolation, all of which diminish the quality of life for the older adult and impose an economic burden on the whole society (6, 7). As reported, healthcare costs associated with GSs have been estimated to reach $164 billion annually in the US and over $182 billion in 18 European nations combined (7, 8). Therefore, GSs pose not only a medical issue but also a significant challenge to public health and socio-economic factors.

Physical activity (PA) has gained increasing attention in recent decades due to its lower cost and higher adherence than traditional strategies like medication, long-term nursing, and hospitalization (9, 10). There is strong evidence that PA was associated with a risk reduction in the incidence of GSs. A previous Longitudinal cohort study found that maintaining a regular frequency of PA is associated with frailty among European community-dwelling older adult (11). Another cross-sectional study conducted in Japan discovered a correlation between increased leisure-time PA and a reduced incidence of dysphagia, suggesting the potential protective role of PA on GSs (12). Conversely, insalubrious lifestyles such as sedentary behavior (SB) in older adults hurt muscle strength (13), bone health (14), and cognitive function (15). Several cohort studies investigating lifestyle factors and risk of GSs reported that SB was independently and positively associated with frailty and falls (16, 17). As two modifiable lifestyles, both PA and SB are strongly associated with several GSs. However, existing observational studies cannot eliminate the potential for reverse causation and confounding factors, hindering the establishment of causal relationships. Previous studies on PA and SB have primarily used self-reported activity measures instead of directly measuring overall mean acceleration with a wrist-worn accelerometer (18). This reliance on self-validated measures introduces the potential for information bias (19, 20). Therefore, the causal relationship between objectively measured PA, SB, and GSs remains uncertain.

Mendelian randomization (MR) study is a statistical method to infer potential causal relationships between exposure and outcome (21). Although MR methods have limitations, such as genetic variants only explaining a portion of the exposure variability and the possibility of unrecognized confounders still existing. An advantage of MR studies is their ability to strengthen result justification by minimizing the impact of confounding factors on result accuracy. Simultaneously, MR studies offer more robust evidence to ascertain the causal relationship between exposure and outcome (22). Therefore, this study used MR to investigate the causal relationship between accelerometer-based PA, SB, and GSs. These findings can potentially contribute novel strategies for preventing, diagnosing, and treating GSs.

## 2 Materials and methods

### 2.1 Study design

**Figure 1:**
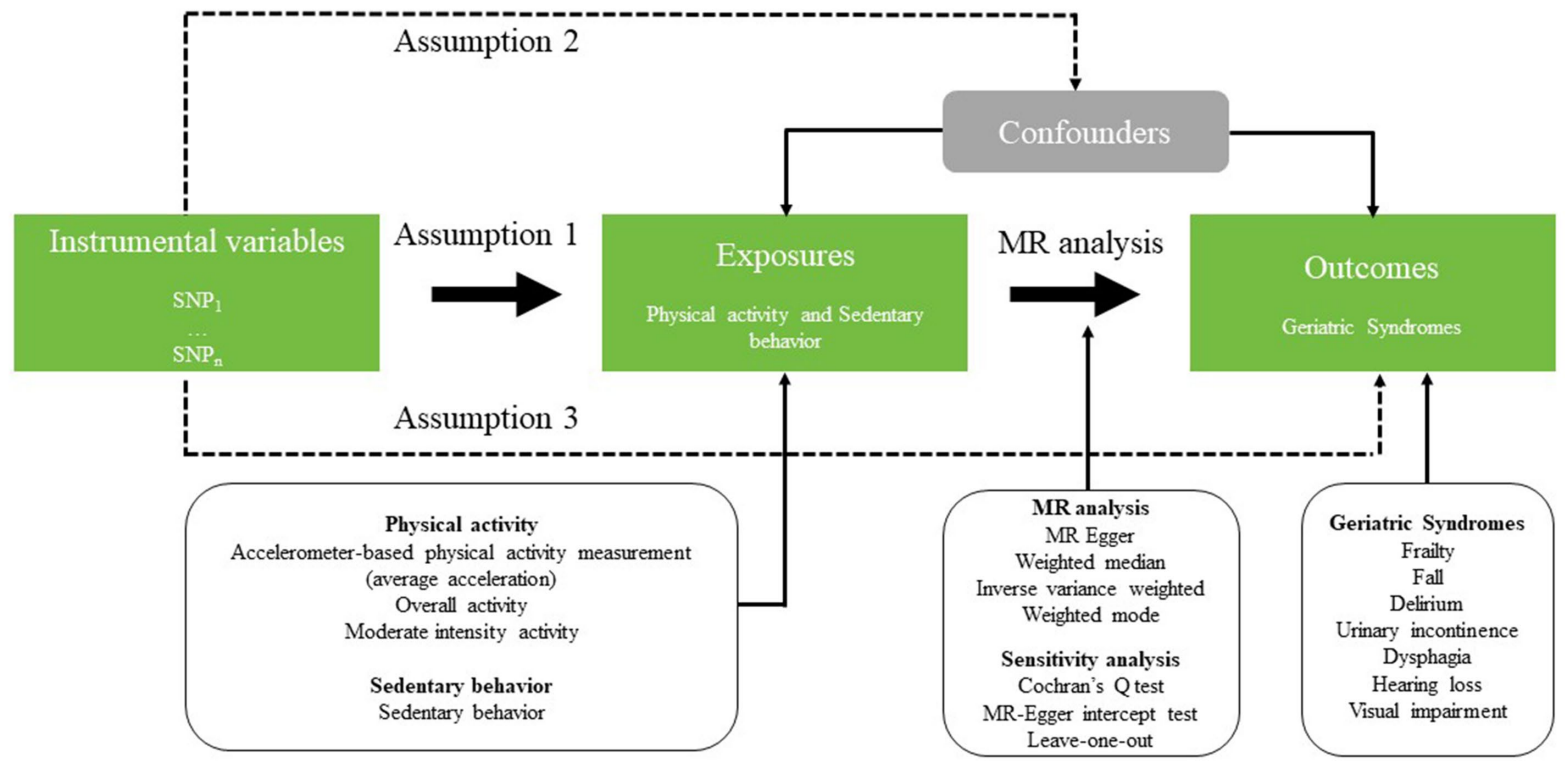
Schematic representation of the three hypotheses and study design.

This study used two-sample MR to analyze the causal relationship between PA, SB, and GSs. A valid MR analysis must be supported by three key assumptions (23): (1) the selected genetic variants as instrumental variables (IVs) are robustly correlations with the exposure (24); (2) there were no unmeasured confounders for associations between genetic variants and outcomes (25); (3) the genetic variants affect the outcome only through their effect on the exposure of interest, that is, there is no horizontal pleiotropy between genetic variants and outcome (26). The overall design was shown in Figure 1.

For this MR study, we prioritized using IVs from Genome-Wide Association Studies (GWAS) databases over those from single-nation databases. This approach was chosen due to several advantages associated with GWAS databases (27): (1) GWAS databases include genetic data from a wide range of populations globally, providing a more comprehensive selection of SNPs. This ensures that our study captures a representative sample of genetic variations; (2) GWAS typically involve large-scale sample collections, resulting in higher statistical power and more reliable findings than smaller, single-nation studies; (3) the inclusion of diverse populations in GWAS helps to minimize regional and ethnic differences, enhancing the validity of our causal inferences.

In addition, we followed the Strengthening the Reporting of Observational Studies in Epidemiology–Mendelian Randomization (STROBE-MR) reporting guidelines in this study (28). All studies contributing data to these analyses obtained institutional review board approval from the respective countries, aligning with the Declaration of Helsinki (29). The present study did not need additional ethical approval since the original studies have received appropriate ethics and institutional review board approval.

### 2.2 Data sources

Three types of accelerometer-based PA and one type of accelerometer-based SB were included in our study as exposures, including acceleration average (AccAve), overall activity, moderate-intensity physical activity (MPA), and SB. GWAS for AccAve came from a recent study on PA among 377,234 participants from the UK Biobank (30). It required participants to wear an Axivity AX3 accelerometer for at least 72 h in a week, data <72 h or not completed every hour of the 24-h cycle, and other outliers were excluded (31). Overall activity, MPA, and SB were from another GWAS on PA and SB measured using wrist-worn accelerometers (*N* = 91,105); participants were asked to wear activity trackers over 7 days (32). Details of PA, SB, and accelerometers are in Supplementary Table S1.

Drawing from the GSs literature, this study focused on seven common GSs as outcomes, including frailty (4), urinary incontinence (2), dysphagia (33), delirium, fall (1), hearing loss and visual impairment (34). The source of GWAS for exposures and outcomes were shown in Table 1, and detailed information on each of the GSs is shown in Supplementary Table S2.

**Table 1:** Characteristics and sources of data in this study.

Trait	Sample size	Population	No. of SNPs	GWAS ID	PMID
Frailty	175,226	European	7,589,717	ebi-a-GCST90020053	34431594
Fall	218,792	European	16,380,466	finn-b-FALLS	/
Delirium	218,792	European	16,380,466	finn-b-ALCODELIRIUM	/
Urinary incontinence	463,010	European	9,851,867	ukb-b-11531	/
Dysphagia	463,010	European	9,851,867	ukb-b-7073	/
Hearing loss	330,759	European	10,858,770	ebi-a-GCST90012115	32986727
Visual impairment	211,769	European	16,380,453	finn-b-H7_BLINDANDVISIMPAIRMENT	/
AccAve	91,084	European	11,796,201	ebi-a-GCST006099	29899525
Overall activity	91,105	European	9,926,106	/	30531941
MPA	91,105	European	9,926,106	/	30531941
SB	91,105	European	9,926,106	/	30531941

It is important to note that this part of the study was time-consuming for both the human subjects and the researchers due to the need for extended wear time and rigorous data collection protocols.

### 2.3 Genetic instrumental variable selection

Inclusion criteria: (1) single nucleotide polymorphisms (SNPs) with complete genetic significance, ensuring independence and high correlation between exposure factors and outcome variables, were chosen as instrumental variables (35). In previous MR studies, accelerometer-based GWAS of PA identified only 2 independent genome-wide significant SNPs with 5–14% heritability of PA (19). Heritability estimates indicate that SNPs, not currently identified as genome-wide significant, may play a role in the variation of PA. Consequently, SNPs were chosen to achieve genome-wide significance (*p* < 5e−06). This approach of adjusting statistical thresholds for genetic tools has been employed in earlier MR studies where physical activity served as an exposure (19); (2) A criterion of *F* > 10 was employed to define a strong association. To evaluate the strength of the instrumental variables (IVs), the F statistic of an individual SNP was computed. If *F* > 10, it indicates a negligible possibility of weak instrumental variable bias (36); (3) to exclude the influence of gene pleiotropy on the results, the linkage disequilibrium coefficient *r*^2^ was set to 0.001, and the width of the linkage disequilibrium region was set to 10,000 kb, to ensure the independence of each SNP (21, 37).

Exclusion criteria: given the close association of various confounding factors with the pathogenesis of sarcopenia, SNPs were scrutinized against the PhenoScanner database^1^ to identify potential violations of independence and exclusivity assumptions. SNPs closely linked to the geriatric syndrome were then excluded. After screening based on inclusion and exclusion criteria, the detailed information on SNPs used as instrumental variables in the MR analyses is shown in Supplementary Tables S3–S6.

### 2.4 Statistical analysis

In this paper, we used the following four different methods to estimate the causal relationship between PA, SB, and GSs: (1) we used the inverse variance weighted (IVW) method as the main analysis, which is essentially a meta-analysis method. IVW assesses causality by meta-analyzing the Wald ratios for each of the included SNP (37, 38); (2) we used MR-Egger to detect several violations of the assumptions of the standard instrumental variables and provide estimates of effects that are unaffected by these violations. MR-Egger also provides a sensitivity analysis for the robustness of the results of MR studies (23); (3) we also used the weighted median approach because this method yields reliable estimates of causal effects, even though <50% of the information comes from the null instrument (25); (4) weighted mode is consistent when the largest subset of instruments which identify the same causal effect are valid instruments, even if the majority of others are invalid (39). The assessment of causality between exposure and outcome was quantified as odds ratios (OR) and their respective 95% confidence intervals (CI). Statistical significance was indicated by a *p* < 0.05.

In addition, we used Cochran’s *Q*-test to detect heterogeneity among the selected SNPs, and if heterogeneity existed (*p* < 0.05) (40). To identify potential pleiotropy, we tested for MR-Egger-intercept horizontal pleiotropy, with a *p*-value for the intercept >0.05 indicating that no horizontal pleiotropy existed (41). Meanwhile, we removed individual SNPs one by one using the leave-one-out method and calculated the meta-effect estimates and confidence intervals for the remaining SNPs (42). It was used to test the effect of individual SNPs on causal inference.

In this study, R software (version 4.0.2) was used for all data analysis as well as for drawing statistical plots, mainly R packages (TwoSampleMR, Pacman, Matrix, and Mendelian randomization) were used for data analysis (43). These packages are free for free on the R Software website.^2^

## 3 Results

### 3.1 MR results

**Figure 2:**
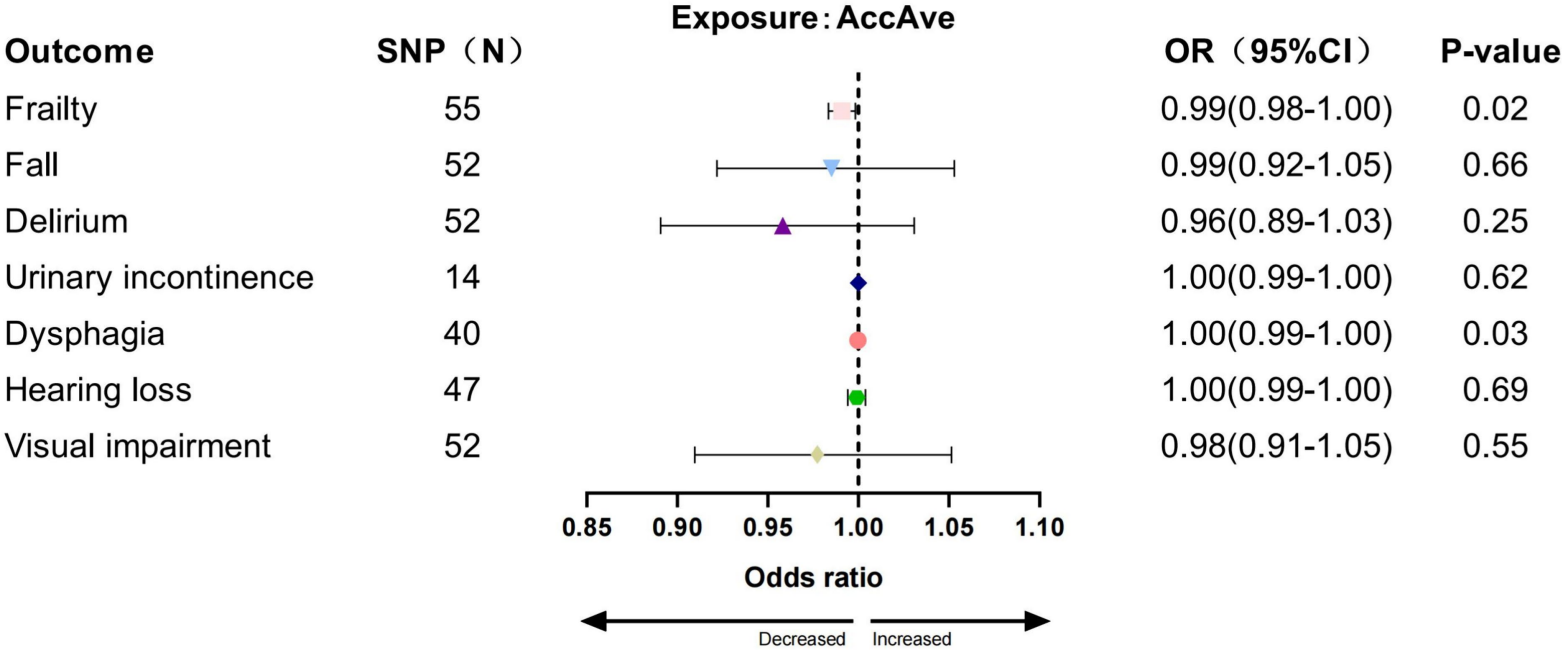
Association between AccAve and GSs by IVW method.

**Figure 3:**
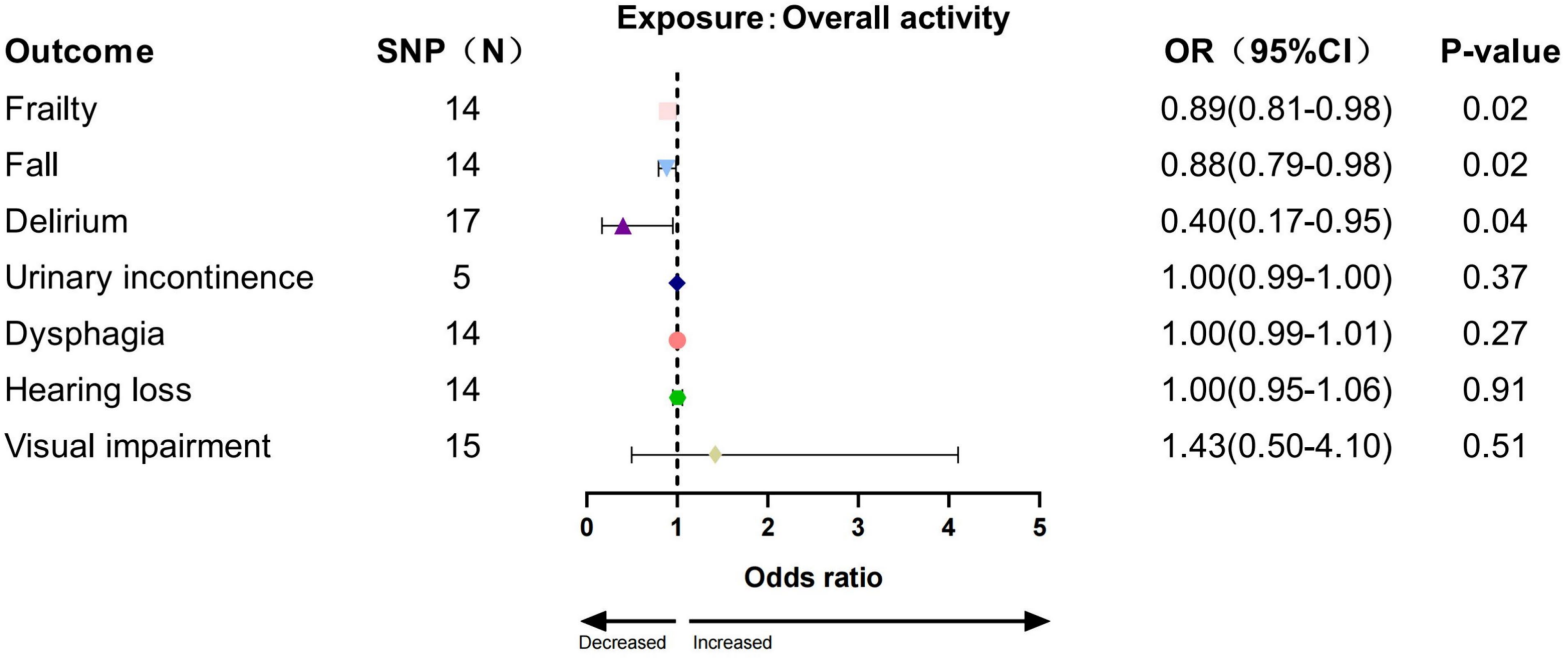
Association between overall activity and geriatric syndromes by IVW method.

**Figure 4:**
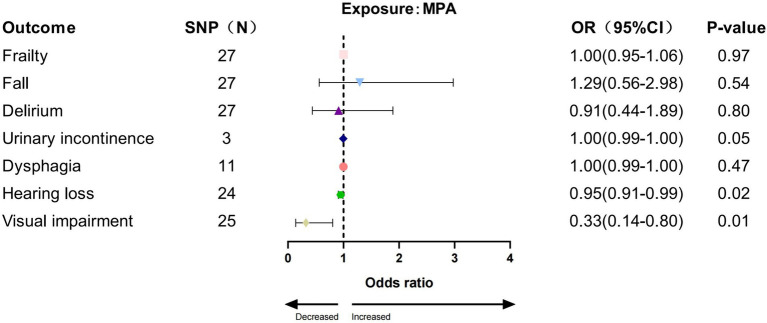
Association between MPA and GSs by IVW method.

In the IVW analysis, we found a significant causal relationship between AccAve and a low risk of two GSs (frailty, OR = 0.99, 95% CI:0.98–0.99, *p* = 0.01; dysphagia, OR = 0.99, 95% CI: 0.99–0.99, *p* = 0.03; Figure 2). The weighted median, weighted mode, and MR-Egger analyses yielded similar patterns of effects (Supplementary Table S7). Similarly, there was a causal relationship between genetically predicted accelerometer-based “overall activity” PA and a low risk of two GSs (frailty, OR = 0.89, 95% CI: 0.81–0.98, *p* = 0.01; delirium, OR = 0.40, 95% CI: 0.16–0.95, *p* = 0.03; Figure 3) according to the IVW method. As shown in Figure 4, there was a significant causal relationship between accelerometer-based MPA and a low risk of three GSs (urinary incontinence, OR = 0.99, 95% CI: 0.99–1.00, *p* = 0.04; hearing loss, OR = 0.94, 95% CI: 0.90–0.99, *p* = 0.02; visual impairment, OR = 0.33, 95% CI: 0.13–0.80, *p* = 0.01).

**Figure 5:**
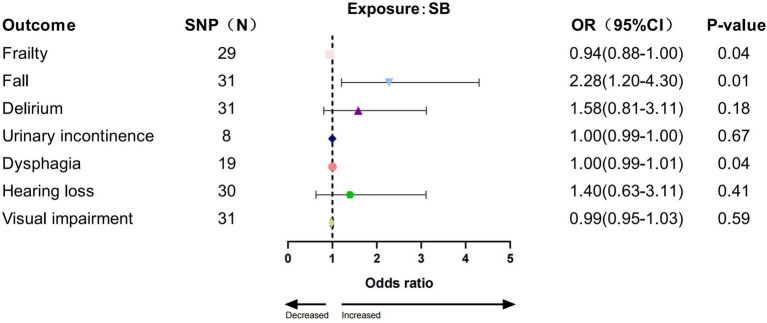
Association between SB and geriatric syndromes by IVW method.

Among the tested SB phenotypes, IVW analysis indicated that accelerometer assessed SB increased the risk for three GSs (frailty: *p* = 0.039, OR = 0.939, 95% CI: 0.885–0.997; fall: *p* = 0.011, OR = 2.28, 95% CI: 1.20–4.30; dysphagia: *p* = 0.044, OR = 1.004, 95% CI: 1.000–1.007, Figure 5). The results from other MR methods showed a consistent direction (Supplementary Table S7).

### 3.2 Sensitivity analysis

To assess the robustness of the above results, a series of sensitivity analyses, including Cochran’s *Q*-test, MR Egger intercept test, and “leave-one-out,” were conducted (Supplementary Table S8). All *p*-values of the MR-Egger intercept tests were > 0.05, indicating that no horizontal pleiotropy existed. Meanwhile, heterogeneity was observed in the Cochran’s *Q-*test between AccAve and Frailty (*Q* = 54, *p* < 0.05). Although heterogeneity was observed in specific findings, it did not render the MR estimations inaccurate as the random-effect IVW method used in this work has the potential to mitigate the overall variability.

In the “leave-one-out” approach (Supplementary Figures S1–S10), each line represents a single nucleotide polymorphism (SNP). Black dots represent the meta-effect estimates obtained when eliminating that particular SNP, while horizontal lines reflect the appropriate confidence intervals. The red line indicates the position of the zero effect. As depicted in the diagram, the total effect estimate remains mostly unchanged when eliminating each SNP. Additionally, all error lines are positioned either to the right or left of zero, indicating a higher level of reliability in the data.

## 4 Discussion

To the best of our knowledge, this is the first MR study to investigate the genetic association of PA, SB, and seven prevalent GSs (frailty, falls, delirium, urinary incontinence, dysphagia, hearing loss, and visual impairment). According to the data shown in the results, genetically predicted accelerometer-based PA (AccAve, overall activity, MPA) was associated with a lower risk of 6 out of 7 GSs (frailty, dysphagia, delirium, urinary incontinence, hearing loss, and visual impairment), and accelerometer-based SB was associated with an increased risk of GSs (frailty, falls, dysphagia). Sensitivity analyses also indicated that results were robust in general. These findings provided a better understanding that PA is, in general, superior to SB in the older adult and had clinical implications for patients and caregivers.

In a longitudinal cohort study involving 1,735 European community-dwelling older adults, regular PA was associated with preserving or enhancing frailty (11). In another prospective, single-center cohort study with 132 participants aged 60 and older, PA was linked to a decreased incidence of delirium, particularly among women (18). Furthermore, a cross-sectional questionnaire-based study investigated the association between dysphagia risk and daily PA, as well as leisure-time exercise, in 3,070 community-dwelling Japanese older adults. The findings revealed that a higher level of leisure-time PA was associated with a reduced risk of dysphagia (12). However, research examining the relationship between PA and the mentioned three GS in older adults has yielded conflicting results. For instance, in a randomized controlled trial with 1,635 participants aged 70–89, the findings indicated that an MPA program did not correlate with a decreased risk of frailty (44). The present study aligns with this perspective. Additionally, a previous study identified a potential causal relationship between MPA and a reduced risk of urinary incontinence (45), hearing loss (46) and visual impairment in older adults (47). Our study, in supporting the aforementioned findings, further substantiates the potential causal relationship for them. Unfortunately, our study did not identify a potential causal relationship between accelerometer-based PA and fall risk. We have access to an explanation for this. In the MR study, SNPs associated with PA were used as instrumental variables to explore whether there is any link between exposure factors and outcome variables (48). This methodology enabled us to clearly and definitively determine the distinct influence of specific genetic variations on these outcomes, without any interference from other intricate elements (49). However, falls are not just attributed to individual genetic variations but also encompass intricate interplays between balance, muscle strength, environmental risk factors, and substance use (50, 51). Therefore, future research should look at the involvement of multiple SNPs and other complex factors in falls to provide a more complete approach to fall prevention in older persons.

There are various potential mechanisms can be used to illustrate the inverse association between PA and GSs. First, PA could induce several neuromuscular adaptations to slow down the aging-related decline in muscle function, which is suspected to be closely linked to the development of GSs (52, 53). Recent studies have shown that PA leads to the increase in peak firing frequencies of motoneurons and drives motor neuron activation, resulting in enhanced performance and function of the motor unit, which is the foundation for maintaining muscle strength in older adults (54, 55). Second, PA provides a healthy anti-inflammatory environment, largely by releasing muscle-derived myokines that accelerate myocardial regeneration and prevent age-related loss (56). Additionally, PA increases the availability of several growth factors to achieve delayed cognitive decline in the older adult and thus plays a protective role in GSs (57). According to reports, memory loss and cognitive impairment could be risk factors for GSs (58). Animal and human studies have shown that PA enhances brain health, and thus cognitive health, by increasing the availability of several growth factors in the neurotrophin family including BDNF, IGF-1, and VEGF (59, 60).

Another significant discovery in this MR study was the causal association between SB and a higher risk of three GSs (frailty, falls, dysphagia), which aligns with several investigations. A cross-sectional study in rural China revealed that older persons who spent 8 or more hours per day being sedentary were more susceptible to frailty than those who spent <4 h per day being sedentary (61). The adjusted analysis in another observational study with 411 participants demonstrated that there were independent associations between SB and frailty (62). Therefore, the older adult needed to enhance their awareness of reducing SB to control the GSs. In addition to frailty, previous observational studies have demonstrated that SB is associated with an increased risk of falls for older adults living in the community (63, 64). Findings from a meta-analysis provided further evidence in favor of this perspective (65). This can be explained by the fact that SB causes muscle weakness (66), reduced bone mass (67), and sarcopenia in older adults, consequently heightening the risk of frailty and falls (68). Furthermore, this study presents new evidence suggesting a possible causative association between SB and dysphagia in older persons. Specifically, we found that SB was related to an increased risk of dysphagia. One possible explanation is that SB leads to progressive weakening of muscles throughout the body, including the laryngeal and neck muscles associated with swallowing (69). This may affect the coordination and efficiency of swallowing in older adults (70).

There are certain strengths of our study. Compared with previous observational studies, we employed an MR study design to assess the causal associations between genetically predicted PA, SB, and GSs (18, 71). This design could minimize the potential biases due to confounding and reverse causality in the observational studies. Another advantage of this study is that the measures of exposures were obtained using accelerometers, which helps to eliminate potential recall and reaction bias (72). Additionally, the population bias was avoided as the populations under study were all individuals of European ancestry.

However, several limitations in our study need to be addressed. First, the sample selection might introduce bias. Our data predominantly come from Europe, which may not fully represent the global population or specific populations. This geographic and demographic limitation could affect the generalizability of our findings. Second, the representativeness of the sample should be considered. Although we employed rigorous random sampling techniques, the inherent variability in genetic backgrounds and environmental exposures across different populations might influence the results. Future studies with more diverse and larger samples are warranted to confirm our findings. Third, accelerometers also possess some constraints. Measuring posture and other types of PA and SB such as inactive, light exercise, and non-ambulatory activity is challenging (73). Therefore, our study focused primarily on overall PA and SB metrics in the sample. Additional research is required to investigate the correlation between various forms of PA, SB, and GSs. Lastly, although we drew on the largest available GWAS, some identified few genome-wide significant SNPs, which could result in relatively weak genetic instruments. To address this, we incorporated thresholds established in previous MR studies, employed additional SNPs as instruments, and performed sensitivity analyses (19, 74, 75). This approach aimed to mitigate the potential influence of alternative thresholds on the outcomes, thus enhancing the reliability of our findings.

## 5 Conclusion

The present study used a genetic approach and revealed that PA (AccAve, overall activity, MPA) was potentially causally associated with a lower risk of some of the six GSs (frailty, dysphagia, delirium, urinary incontinence, hearing loss, and visual impairment), whereas the accelerometer-based SB was potentially causally associated with a greater risk of three GSs (frailty, falls, and dysphagia). Overall, this study supports the hypothesis that strengthening PA and reducing SB are effective strategies for reducing GSs.

## Data availability statement

The original contributions presented in the study are included in the article/Supplementary material, further inquiries can be directed to the corresponding author.

## Ethics statement

Ethical approval was not required for the study involving humans in accordance with the local legislation and institutional requirements. Written informed consent to participate in this study was not required from the participants or the participants' legal guardians/next of kin in accordance with the national legislation and the institutional requirements.

## Author contributions

JC: Conceptualization, Data curation, Formal analysis, Methodology, Resources, Software, Supervision, Validation, Visualization, Writing – original draft, Writing – review & editing. YL: Writing – original draft, Supervision. JY: Writing – original draft, Supervision. XZ: Writing – original draft, Validation, Supervision. YP: Writing – original draft, Methodology, Project administration.

## Footnotes

^1^ http://www.phenoscanner.medschl.cam.ac.uk/

^2^ https://www.r-project.org/
