# Supplementary material for: The relationship between accelerometer-based physical activity, sedentary behavior, and seven common geriatric syndromes: a two-sample Mendelian randomization study
Source: Front Public Health. 2024 Aug 5;12:1406303. doi: 10.3389/fpubh.2024.1406303 (PMC11330792; doi:10.3389/fpubh.2024.1406303)
Supplement: Supplementary file 1 [file Data_Sheet_1.docx]

# Supplementary Materials

Figure S1. Leave-one-out sensitivity analysis for the causal effect of AccAve on frailty

Figure S2. Leave-one-out sensitivity analysis for the causal effect of AccAve on dysphagia

Figure S3. Leave-one-out sensitivity analysis for the causal effect of overall activity on frailty

Figure S4. Leave-one-out sensitivity analysis for the causal relationship between overall activity and delirium

Figure S5. Leave-one-out sensitivity analysis for the causal effect of MPA on urinary incontinence

Figure S6. Leave-one-out sensitivity analysis for the causal effect of MPA on hearing loss

Figure S7. Leave-one-out sensitivity analysis for the causal effect of MPA on visual impairment

Figure S8. Leave-one-out sensitivity analysis for the causal effect of SB on frailty

Figure S9. Leave-one-out sensitivity analysis for the causal relationship between SB and falls

Figure S10. Leave-one-out sensitivity analysis for the causal effect of SB on dysphagia

Table S1. Detailed information on the accelerometers included in the study.

Table S2. Detailed information on the GSs included in the study.

Table S3. Detailed information on the SNPs used as instrumental variables in the MR analyses of AccAve in relation to GSs.

Table S4. Detailed information on the SNPs used as instrumental variables in the MR analyses of overall activity in relation to GSs.

Table S5. Detailed information on the SNPs used as instrumental variables in the MR analyses of MPA in relation to GSs.

Table S6. Detailed information on the SNPs used as instrumental variables in the MR analyses of SB in relation to GSs.

Table S7. MR results of the association between PA, SB and GSs.

Table S8. Tests for horizontal pleiotropy and heterogeneity.


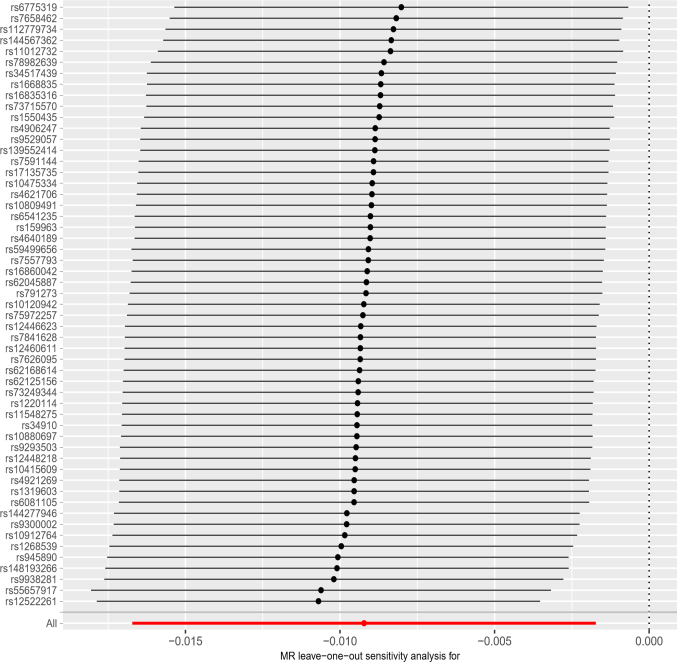


**Figure S1.** Leave-one-out sensitivity analysis for the causal effect of AccAve on frailty


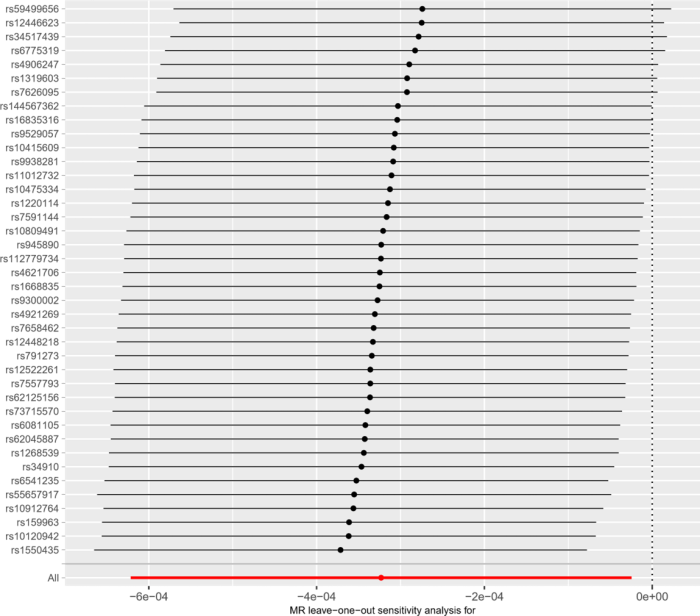


**Figure S2.** Leave-one-out sensitivity analysis for the causal effect of AccAve on dysphagia

All

rs12595220

rs78723158

rs13270182

rs283053

rs117334682

rs11643658

rs2073056

rs35405235

rs4445878

rs7548269

rs764361

rs11526062

rs1290367

rs2058844

−0.20

−0.15

−0.10

−0.05

0.00

MR Leave−one−out sensitivity analysis for

**Figure S3**. Leave-one-out sensitivity analysis for the causal effect of overall activity on frailty


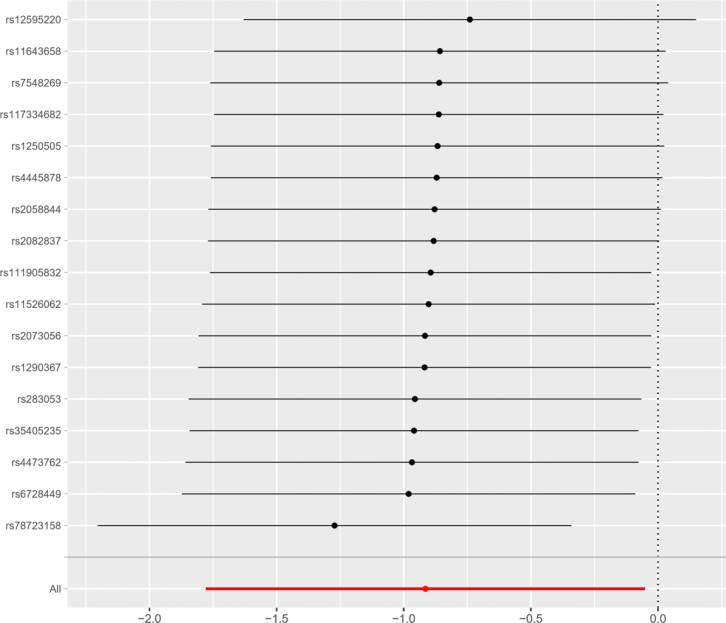


**Figure S4.** Leave-one-out sensitivity analysis for the causal relationship between overall activity and delirium


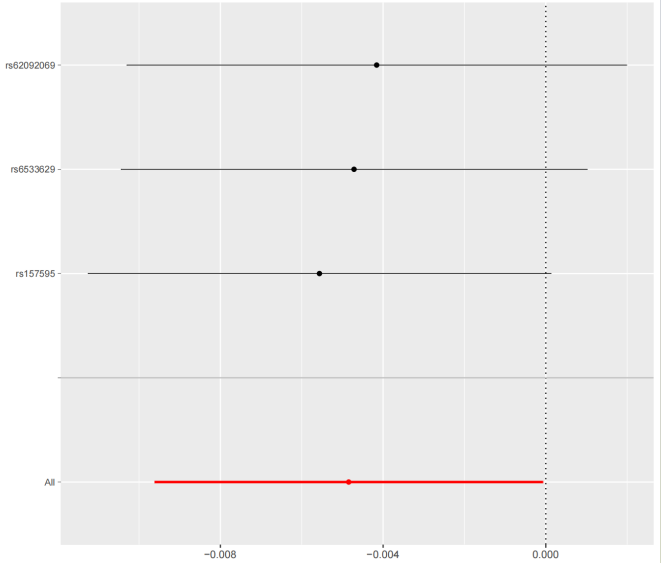


**Figure S5.** Leave-one-out sensitivity analysis for the causal effect of MPA on urinary incontinence


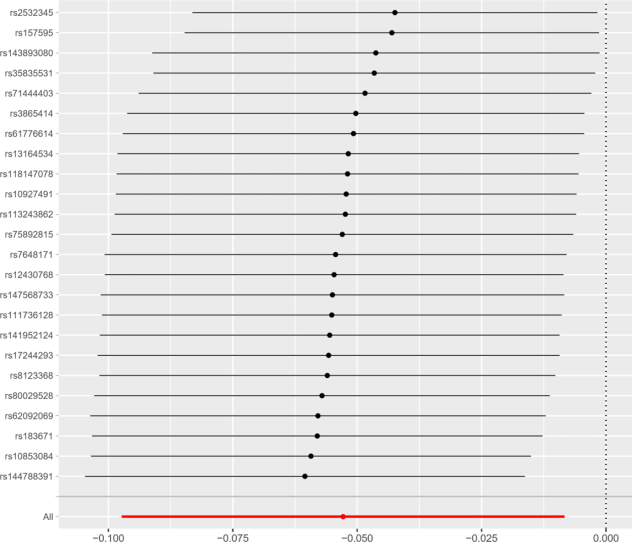


**Figure S6.** Leave-one-out sensitivity analysis for the causal effect of MPA on hearing loss


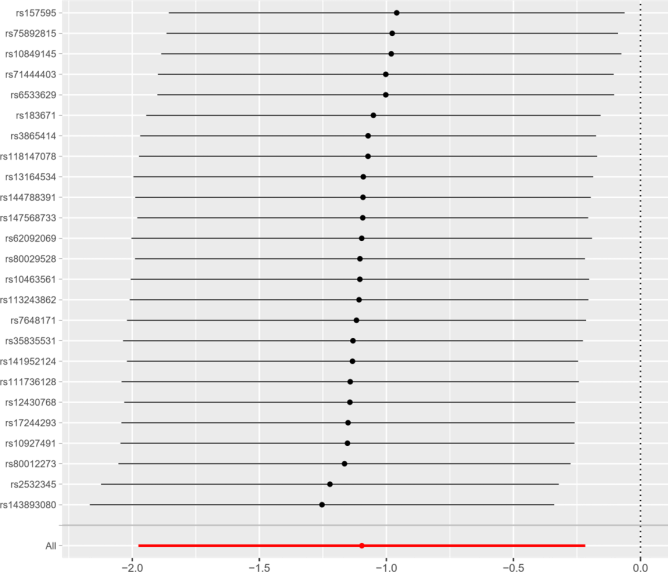


**Figure S7.** Leave-one-out sensitivity analysis for the causal effect of MPA on visual impairment


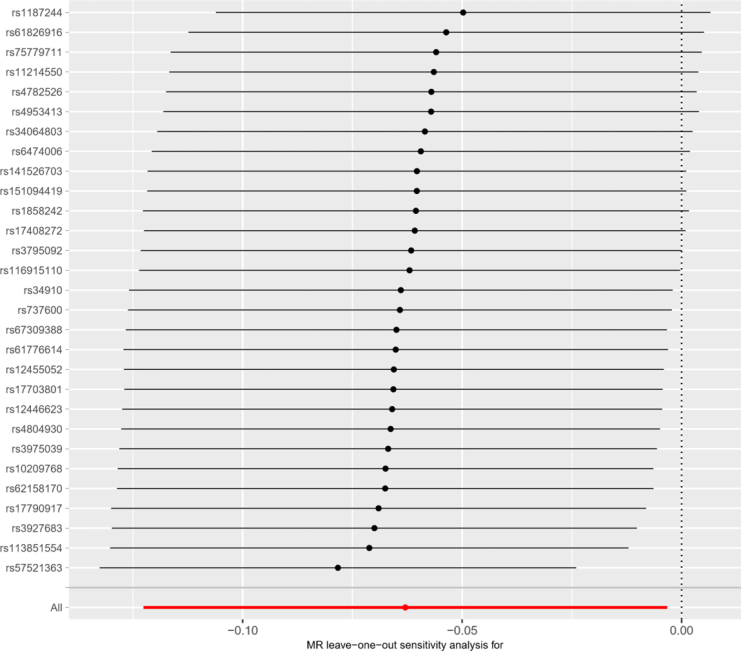


**Figure S8.** Leave-one-out sensitivity analysis for the causal effect of SB on frailty


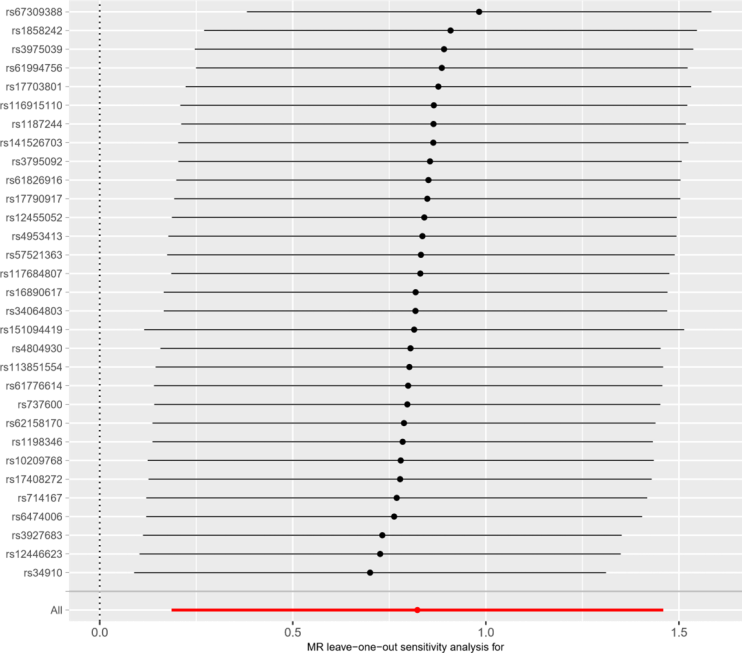


**Figure S9.** Leave-one-out sensitivity analysis for the causal relationship between SB and falls


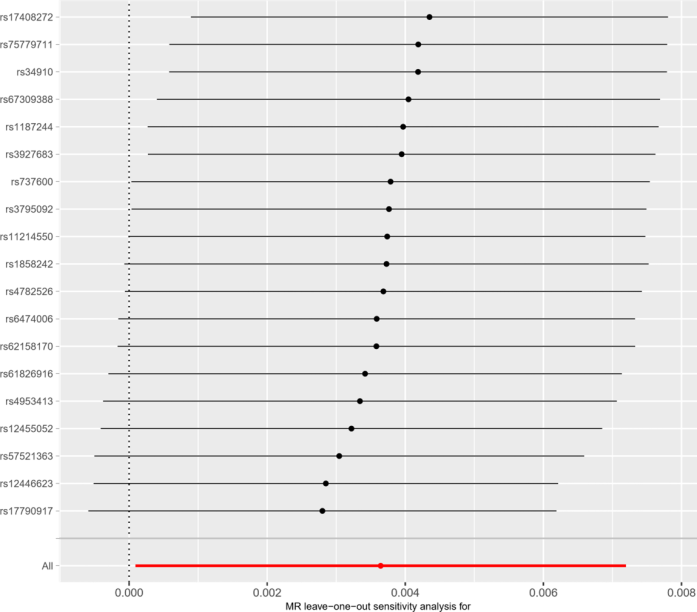


**Figure S10.** Leave-one-out sensitivity analysis for the causal effect of SB on dysphagia

| **Trait** | **Wearable device annotation** | **Device wear time** | **Definition of trait** | **Acceleration vector magnitude (mg)** |
| --- | --- | --- | --- | --- |
| AccAve | Axivity AX3 wrist-worn accelerometer | 7 days,24 hours per day | The seven-day average of the total acceleration of the wearer's accelerometer. | / |
| Overall activity | Axivity AX3 wrist-worn triaxial accelerometer | 7 days,24 hours per day | Total seven-day value of the accelerometer. | / |
| MPA | Axivity AX3 wrist-worn triaxial accelerometer | 7 days,24 hours per day | Moderate-intensity activities are behaviours that have a MET (metabolic equivalent of task) range of 3 to 6 and are dominated by household activities and leisure and physical activities. | 225 - 425 |
| SB | Axivity AX3 wrist-worn triaxial accelerometer | 7 days,24 hours per day | A sedentary behaviour is one which has a MET(Metabolic Equivalent of Task) energy expenditure score of≤ 1.5 and occurs in asitting, lying, or reclining posture | < 25 |

**Table S1**.Detailed information on the accelerometers included in the study.

**Table S2.**Detailed information on the GSs included in the study.

| **GSs** | **Definition** | **Assessments** | **Prevalence** |
| --- | --- | --- | --- |
| Frailty | Frailty is theoretically defined as a clinically recognizable state of increased vulnerability resulting from aging-associated decline in reserve and function across multiple physiologic systems such that the ability to cope with everyday or acute stressors is comprised. | (1): Frailty phenotype;  (2): Frailty index. | approximately 11% |
| Falls | Falls in the outpatient setting are usually defined as “coming to rest unintentionally on the ground or lower level, not due to an acute overwhelming event”6 (e.g., stroke, seizure, loss of consciousness) or external event to which any person would be susceptible. | STEADI (Stopping Elderly Accidents, Deaths, and Injuries) algorithm. | 30% - 40% |
| Delirium | Delirium is defined as an acute disturbance in attention and cognition that develops over a short period of time. | (1): Delirium Observation Screening Scale;  (2): The Nursing Delirium Screening Checklist;  (3): The Neelon and Champagne (NEE-CHAM) Confusion Scale. | 8% - 17% |
| Urinary incontinence | Urinary incontinence may be defined as the complaint of any involuntary loss of urine. | (1): past medical history;  (2): physical examination;  (3): urinalysis. | 7% - 42% |
| Dysphagia | Dysphagia is a common type of dysphagia referring to a disturbance in the oral preparatory, oral and/or pharyngeal swallowing phases. | (1): past medical history;  (2): physical examination;  (3): oral motor examination;  (4): assessment of food intake. | 6% - 50% |
| Hearing loss | age-related hearing impairment (ARHI),  also known as presbycusis. | (1): self-reported hearing impairment;  (2): 10-item Hearing Handicap Inventory. | 25% - 30% |
| Visual impairment | Vision loss is a major global health concern among older adults, as the prevalence of vision loss increases rapidly with age. | (1): self-report questionnaires | approximately 50% |

**Table S3.** Detailed information on the SNPs used as instrumental variables in the MR analyses of AccAve in relation to GSs.

| **beta.exposure** | **se.exposure** | **eaf.exposure** | **SNP** | **pval.exposure** | **F** |
| --- | --- | --- | --- | --- | --- |
| -0.17 | 0.03 | 0.57 | rs159963 | 3.07E-06 | 1342.04 |
| 0.23 | 0.04 | 0.21 | rs16835316 | 5.11E-08 | 1801.54 |
| 0.53 | 0.11 | 0.02 | rs11548275 | 3.81E-06 | 1291.95 |
| -0.30 | 0.05 | 0.12 | rs34517439 | 4.38E-08 | 1873.98 |
| 0.22 | 0.04 | 0.25 | rs791273 | 1.12E-07 | 1724.03 |
| -0.20 | 0.04 | 0.24 | rs10912764 | 1.69E-06 | 1394.79 |
| -0.16 | 0.03 | 0.42 | rs1319603 | 4.39E-06 | 1283.78 |
| -0.25 | 0.05 | 0.14 | rs6541235 | 7.33E-07 | 1481.67 |
| -0.19 | 0.03 | 0.29 | rs62168614 | 6.03E-07 | 1506.92 |
| -0.19 | 0.04 | 0.25 | rs1220114 | 4.84E-06 | 1327.00 |
| 0.20 | 0.04 | 0.22 | rs7557793 | 4.28E-06 | 1292.09 |
| 0.17 | 0.03 | 0.38 | rs7591144 | 3.15E-06 | 1310.76 |
| 0.18 | 0.04 | 0.27 | rs34910 | 4.47E-06 | 1291.73 |
| -0.22 | 0.04 | 0.72 | rs6775319 | 3.46E-08 | 1859.36 |
| 0.28 | 0.06 | 0.09 | rs16860042 | 3.05E-06 | 1333.60 |
| 0.17 | 0.03 | 0.43 | rs7626095 | 2.19E-06 | 1404.13 |
| -0.23 | 0.04 | 0.17 | rs7658462 | 1.16E-06 | 1444.90 |
| 0.21 | 0.04 | 0.18 | rs73249344 | 2.90E-06 | 1340.20 |
| -0.24 | 0.05 | 0.12 | rs10475334 | 4.53E-06 | 1281.80 |
| -0.32 | 0.05 | 0.11 | rs9293503 | 2.08E-08 | 1995.68 |
| -0.33 | 0.07 | 0.07 | rs17135735 | 1.56E-06 | 1406.58 |
| -0.21 | 0.03 | 0.34 | rs12522261 | 3.88E-08 | 1857.68 |
| 0.17 | 0.03 | 0.61 | rs4921269 | 2.66E-06 | 1341.25 |
| -0.56 | 0.12 | 0.02 | rs144277946 | 4.53E-06 | 1323.33 |
| 0.20 | 0.04 | 0.71 | rs945890 | 2.15E-07 | 1654.74 |
| 0.20 | 0.04 | 0.72 | rs144567362 | 3.89E-06 | 1616.29 |
| -0.16 | 0.03 | 0.54 | rs4621706 | 3.74E-06 | 1312.75 |
| 0.26 | 0.05 | 0.11 | rs7841628 | 4.59E-06 | 1275.07 |
| 0.20 | 0.04 | 0.22 | rs73715570 | 3.26E-06 | 1343.22 |
| -0.20 | 0.04 | 0.25 | rs10809491 | 1.43E-06 | 1419.50 |
| -0.17 | 0.03 | 0.56 | rs10120942 | 2.62E-06 | 1341.75 |
| 0.18 | 0.03 | 0.41 | rs1268539 | 2.73E-07 | 1610.75 |
| -0.22 | 0.03 | 0.33 | rs11012732 | 5.41E-09 | 2092.56 |
| 0.17 | 0.03 | 0.42 | rs9300002 | 2.13E-06 | 1373.80 |
| 0.51 | 0.09 | 0.04 | rs148193266 | 3.06E-08 | 1980.27 |
| 0.20 | 0.03 | 0.33 | rs10880697 | 1.22E-07 | 1724.99 |
| -0.88 | 0.17 | 0.01 | rs78982639 | 2.79E-07 | 1628.94 |
| -0.31 | 0.06 | 0.08 | rs75972257 | 1.38E-06 | 1459.24 |
| 0.17 | 0.03 | 0.47 | rs9529057 | 1.71E-06 | 1397.47 |
| -0.17 | 0.03 | 0.65 | rs4906247 | 4.53E-06 | 1280.63 |
| 0.19 | 0.04 | 0.24 | rs62045887 | 4.00E-06 | 1287.36 |
| 0.55 | 0.11 | 0.02 | rs139552414 | 3.69E-06 | 1303.96 |
| -0.19 | 0.03 | 0.42 | rs1550435 | 4.99E-08 | 1818.04 |
| 0.18 | 0.03 | 0.33 | rs12448218 | 1.00E-06 | 1463.21 |
| 0.19 | 0.03 | 0.52 | rs9938281 | 1.45E-07 | 1712.49 |
| 0.24 | 0.05 | 0.14 | rs12446623 | 2.21E-06 | 1374.73 |
| 0.18 | 0.03 | 0.47 | rs11865683 | 4.38E-07 | 1553.20 |
| 0.35 | 0.07 | 0.06 | rs4640189 | 1.78E-06 | 1402.00 |
| 0.30 | 0.04 | 0.22 | rs55657917 | 5.02E-12 | 2976.53 |
| 0.19 | 0.03 | 0.31 | rs1668835 | 4.76E-07 | 1556.90 |
| 0.22 | 0.03 | 0.34 | rs59499656 | 2.43E-09 | 2194.79 |
| -0.20 | 0.04 | 0.21 | rs62125156 | 4.54E-06 | 1288.27 |
| -0.18 | 0.03 | 0.32 | rs12460611 | 1.35E-06 | 1436.11 |
| -0.26 | 0.05 | 0.87 | rs10415609 | 1.71E-06 | 1395.72 |
| 0.18 | 0.03 | 0.60 | rs6081105 | 8.21E-07 | 1489.10 |
| -0.18 | 0.03 | 0.30 | rs112779734 | 1.37E-06 | 1416.85 |

**Table S4.** Detailed information on the SNPs used as instrumental variables in the MR analyses of overall activity in relation to GSs.

| **SNP** | **beta.exposure** | **se.exposure** | **pval.exposure** |
| --- | --- | --- | --- |
| rs2082837 | -0.02 | 0.00 | 3.24E-06 |
| rs142804168 | -0.04 | 0.00 | 4.65E-08 |
| rs143769438 | 0.08 | 0.01 | 6.78E-06 |
| rs587626951 | -0.04 | 0.00 | 1.79E-06 |
| rs7823741 | 0.02 | 0.00 | 2.95E-06 |
| rs11526062 | 0.02 | 0.00 | 2.27E-06 |
| rs28661646 | 0.02 | 0.00 | 3.78E-06 |
| rs12595220 | 0.02 | 0.00 | 1.52E-06 |
| rs11643658 | -0.02 | 0.00 | 6.49E-06 |
| rs1290367 | 0.02 | 0.00 | 9.33E-06 |
| rs78723158 | -0.11 | 0.02 | 6.39E-07 |
| rs4473762 | 0.02 | 0.00 | 4.84E-07 |
| rs6728449 | -0.02 | 0.00 | 8.09E-07 |
| rs1250505 | -0.02 | 0.00 | 6.98E-07 |
| rs35405235 | -0.02 | 0.00 | 3.80E-06 |
| rs7548269 | -0.02 | 0.00 | 1.65E-08 |
| rs111905832 | 0.08 | 0.01 | 3.40E-06 |
| rs117334682 | -0.07 | 0.01 | 1.68E-06 |
| rs2058844 | 0.02 | 0.00 | 3.10E-06 |
| rs61149527 | -0.03 | 0.00 | 3.65E-06 |
| rs764361 | -0.04 | 0.00 | 5.80E-07 |
| rs283053 | -0.02 | 0.00 | 2.44E-06 |
| rs13270182 | 0.02 | 0.00 | 5.16E-06 |
| rs4445878 | 0.02 | 0.00 | 3.31E-06 |
| rs2073056 | -0.02 | 0.00 | 1.05E-06 |

**Table S5.** Detailed information on the SNPs used as instrumental variables in the MR analyses of MPA in relation to GSs.

| **SNP** | **beta.exposure** | **se.exposure** | **pval.exposure** |
| --- | --- | --- | --- |
| rs71444403 | 0.04 | 0.01 | 3.77E-06 |
| rs141952124 | 0.07 | 0.01 | 1.55E-06 |
| rs77396736 | -0.02 | 0.00 | 1.36E-06 |
| rs7648171 | 0.02 | 0.00 | 5.52E-07 |
| rs35835531 | 0.03 | 0.00 | 3.84E-06 |
| rs17244293 | 0.05 | 0.01 | 1.96E-07 |
| rs10463561 | 0.02 | 0.00 | 9.80E-07 |
| rs147568733 | -0.07 | 0.01 | 2.10E-07 |
| rs138677488 | 0.03 | 0.00 | 5.32E-06 |
| rs143893080 | 0.09 | 0.02 | 2.02E-06 |
| rs113243862 | -0.06 | 0.01 | 3.61E-06 |
| rs76187114 | 0.05 | 0.01 | 2.26E-06 |
| rs2764021 | -0.02 | 0.00 | 1.46E-06 |
| rs12430768 | 0.04 | 0.01 | 1.37E-05 |
| rs62092069 | -0.02 | 0.00 | 7.16E-08 |
| rs3865414 | 0.02 | 0.00 | 1.08E-05 |
| rs144788391 | -0.07 | 0.01 | 1.19E-06 |
| rs80012273 | -0.04 | 0.00 | 2.52E-06 |
| rs111736128 | -0.06 | 0.01 | 2.58E-06 |
| rs183671 | 0.08 | 0.01 | 5.18E-07 |
| rs143893080 | 0.09 | 0.02 | 2.02E-06 |
| rs6533629 | -0.02 | 0.00 | 2.18E-06 |
| rs367906650 | -0.02 | 0.00 | 1.61E-06 |
| rs13164534 | -0.02 | 0.00 | 2.36E-06 |
| rs10849145 | 0.03 | 0.00 | 1.47E-08 |
| rs10849145 | 0.03 | 0.00 | 1.47E-08 |
| rs118147078 | -0.07 | 0.01 | 3.39E-06 |
| rs80029528 | -0.04 | 0.00 | 5.00E-07 |
| rs75892815 | 0.07 | 0.01 | 5.41E-06 |
| rs157595 | -0.02 | 0.00 | 3.01E-06 |
| rs2532345 | 0.02 | 0.00 | 4.31E-06 |
| rs8123368 | -0.04 | 0.01 | 4.59E-06 |
| rs61776614 | -0.04 | 0.00 | 1.21E-06 |
| rs10853084 | 0.02 | 0.00 | 4.69E-06 |
| rs10927491 | 0.02 | 0.00 | 7.83E-06 |

**Table S6.** Detailed information on the SNPs used as instrumental variables in the MR analyses of SB in relation to GSs.

| **SNP** | **beta.exposure** | **se.exposure** | **pval.exposure** |
| --- | --- | --- | --- |
| rs3927683 | 0.02 | 0.00 | 6.08E-06 |
| rs12741347 | -0.02 | 0.00 | 1.06E-06 |
| rs17379561 | -0.03 | 0.00 | 1.24E-07 |
| rs61826916 | -0.02 | 0.00 | 1.55E-06 |
| rs10916119 | 0.03 | 0.00 | 3.19E-07 |
| rs4953413 | 0.02 | 0.00 | 1.78E-07 |
| rs113851554 | -0.04 | 0.01 | 5.44E-06 |
| rs62158170 | 0.02 | 0.00 | 1.38E-06 |
| rs17408272 | 0.02 | 0.00 | 8.96E-07 |
| rs10209768 | -0.07 | 0.01 | 3.30E-06 |
| rs34910 | 0.02 | 0.00 | 2.96E-07 |
| rs1858242 | 0.03 | 0.00 | 3.76E-09 |
| rs17790917 | -0.03 | 0.00 | 1.51E-07 |
| rs732550 | -0.02 | 0.00 | 3.11E-06 |
| rs737600 | -0.02 | 0.00 | 2.17E-07 |
| rs26579 | 0.02 | 0.00 | 2.62E-09 |
| rs25981 | 0.02 | 0.00 | 2.73E-09 |
| rs6870096 | -0.02 | 0.00 | 2.39E-08 |
| rs16890617 | -0.03 | 0.00 | 3.65E-06 |
| rs116915110 | 0.04 | 0.01 | 1.06E-05 |
| rs189695099 | 0.09 | 0.02 | 3.34E-06 |
| rs11487196 | -0.02 | 0.00 | 1.43E-05 |
| rs34858520 | 0.02 | 0.00 | 4.48E-09 |
| rs17703801 | 0.04 | 0.01 | 1.13E-05 |
| rs6474006 | 0.02 | 0.00 | 3.01E-06 |
| rs34064803 | 0.03 | 0.00 | 4.79E-06 |
| rs10887455 | -0.02 | 0.00 | 2.75E-06 |
| rs141526703 | 0.06 | 0.01 | 9.18E-06 |
| rs9548984 | 0.02 | 0.00 | 1.53E-06 |
| rs7317533 | -0.03 | 0.00 | 8.00E-06 |
| rs61994756 | -0.10 | 0.02 | 7.04E-06 |
| rs75779711 | -0.03 | 0.00 | 2.48E-07 |
| rs12446623 | 0.03 | 0.00 | 1.38E-06 |
| rs4782526 | -0.02 | 0.00 | 2.94E-06 |
| rs1187244 | 0.02 | 0.00 | 5.46E-07 |
| rs12455052 | -0.02 | 0.00 | 2.30E-06 |
| rs4804930 | -0.04 | 0.00 | 1.09E-06 |
| rs3975039 | -0.05 | 0.01 | 2.80E-06 |
| rs3795092 | -0.02 | 0.00 | 6.16E-06 |
| rs714167 | 0.06 | 0.01 | 4.75E-07 |
| rs151094419 | -0.09 | 0.02 | 4.60E-06 |
| rs117684807 | -0.08 | 0.01 | 7.97E-06 |
| rs1198346 | 0.02 | 0.00 | 1.43E-06 |
| rs4804930 | -0.04 | 0.00 | 1.09E-06 |
| rs67309388 | 0.02 | 0.00 | 2.26E-06 |
| rs61776614 | -0.04 | 0.00 | 1.21E-06 |
| rs11214550 | -0.02 | 0.00 | 3.07E-06 |
| rs57521363 | 0.02 | 0.00 | 6.87E-07 |

**Table S7.** MR results of the association between PA, SB and GSs.

| Exposure | Outcome | method | nsnp | b | se | pval | OR | or_lci95 | or_uci95 |
| --- | --- | --- | --- | --- | --- | --- | --- | --- | --- |
| AccAve | frailty | MR Egger | 55 | -0.00 | 0.01 | 0.54 | 0.99 | 0.96 | 1.02 |
|  |  | Weighted median | 55 | -0.00 | 0.00 | 0.10 | 0.99 | 0.98 | 1.00 |
|  |  | Inverse variance weighted | 55 | -0.00 | 0.00 | 0.01 | 0.99 | 0.98 | 0.99 |
|  |  | Weighted mode | 55 | -0.00 | 0.01 | 0.88 | 0.99 | 0.97 | 1.01 |
|  | dysphagia | MR Egger | 40 | -0.00 | 0.00 | 0.15 | 0.99 | 0.99 | 1.00 |
|  |  | Weighted median | 40 | -0.00 | 0.00 | 0.23 | 0.99 | 0.99 | 1.00 |
|  |  | Inverse variance weighted | 40 | -0.00 | 0.00 | 0.03 | 0.99 | 0.99 | 0.99 |
|  |  | Weighted mode | 40 | 1.97E-05 | 0.00 | 0.96 | 1.00 | 0.99 | 1.00 |
| Overall activity | frailty | MR Egger | 14 | -0.01 | 0.15 | 0.93 | 0.98 | 0.72 | 1.33 |
|  |  | Weighted median | 14 | -0.04 | 0.05 | 0.45 | 0.95 | 0.85 | 1.07 |
|  |  | Inverse variance weighted | 14 | -0.11 | 0.04 | 0.01 | 0.89 | 0.81 | 0.97 |
|  |  | Weighted mode | 14 | -0.02 | 0.10 | 0.81 | 0.97 | 0.79 | 1.19 |
|  | delirium | MR Egger | 17 | 0.93 | 1.30 | 0.48 | -1.61 | 0.19 | 32.63 |
|  |  | Weighted median | 17 | -1.06 | 0.59 | 0.07 | -2.23 | 0.10 | 1.11 |
|  |  | Inverse variance weighted | 17 | -0.91 | 0.44 | 0.03 | -1.77 | 0.16 | 0.95 |
|  |  | Weighted mode | 17 | -1.49 | 1.07 | 0.18 | -3.61 | 0.02 | 1.85 |
| MPA | urinary incontinence | MR Egger | 3 | -0.01 | 0.03 | 0.73 | 0.98 | 0.91 | 1.05 |
|  |  | Weighted median | 3 | -0.00 | 0.00 | 0.06 | 0.99 | 0.98 | 1.00 |
|  |  | Inverse variance weighted | 3 | -0.00 | 0.00 | 0.04 | 0.99 | 0.99 | 0.99 |
|  |  | Weighted mode | 3 | -0.00 | 0.00 | 0.24 | 0.99 | 0.98 | 1.00 |
|  | hearing loss | MR Egger | 24 | 0.01 | 0.05 | 0.72 | 1.01 | 0.92 | 1.12 |
|  |  | Weighted median | 24 | -0.01 | 0.02 | 0.55 | 0.98 | 0.92 | 1.04 |
|  |  | Inverse variance weighted | 24 | -0.05 | 0.02 | 0.01 | 0.94 | 0.90 | 0.99 |
|  |  | Weighted mode | 24 | -0.00 | 0.05 | 0.90 | 0.99 | 0.89 | 1.09 |
|  | visual impairment | MR Egger | 25 | -0.54 | 1.09 | 0.62 | 0.57 | 0.06 | 4.91 |
|  |  | Weighted median | 25 | -0.95 | 0.61 | 0.12 | 0.38 | 0.11 | 1.29 |
|  |  | Inverse variance weighted | 25 | -1.09 | 0.44 | 0.01 | 0.33 | 0.13 | 0.80 |
|  |  | Weighted mode | 25 | -0.89 | 1.04 | 0.39 | 0.40 | 0.05 | 3.14 |
| SB | frailty | MR Egger | 29 | 0.02 | 0.10 | 0.80 | 1.02 | 0.83 | 1.25 |
|  |  | Weighted median | 29 | -0.03 | 0.03 | 0.35 | 0.96 | 0.89 | 1.04 |
|  |  | Inverse variance weighted | 29 | -0.06 | 0.03 | 0.03 | 0.93 | 0.88 | 0.99 |
|  |  | Weighted mode | 29 | -0.00 | 0.07 | 0.93 | 0.99 | 0.85 | 1.15 |
|  | fall | MR Egger | 31 | 0.21 | 0.80 | 0.78 | 1.24 | 0.25 | 6.01 |
|  |  | Weighted median | 31 | 0.69 | 0.47 | 0.13 | 2.00 | 0.79 | 5.04 |
|  |  | Inverse variance weighted | 31 | 0.82 | 0.32 | 0.01 | 2.27 | 1.20 | 4.30 |
|  |  | Weighted mode | 31 | 0.47 | 0.70 | 0.50 | 1.61 | 0.40 | 6.43 |
|  | dysphagia | MR Egger | 19 | 0.00 | 0.01 | 0.66 | 1.00 | 0.97 | 1.03 |
|  |  | Weighted median | 19 | 0.00 | 0.00 | 0.32 | 1.00 | 0.99 | 1.00 |
|  |  | Inverse variance weighted | 19 | 0.00 | 0.00 | 0.04 | 1.00 | 1.00 | 1.00 |
|  |  | Weighted mode | 19 | 0.00 | 0.00 | 0.73 | 1.00 | 0.99 | 1.01 |

**Table S8.**Tests for horizontal pleiotropy and heterogeneity.

| **Exposure** | **Outcome** | **IVs** | **Horizontal pleiotropy** | | | **Heterogeneity** | |
| --- | --- | --- | --- | --- | --- | --- | --- |
|  |  |  | **Egger intercept** | **SE** | **P** | **Cochran's Q** | **P** |
| AccAve | Frailty | 55 | -1.75E-05 | 0.00 | 0.99 | 54 | 4.01E-08 |
|  | Fall | 52 | 0.04 | 0.02 | 0.14 | 51 | 0.26 |
|  | Delirium | 52 | -0.01 | 0.03 | 0.62 | 51 | 0.04 |
|  | Urinary incontinence | 14 | 0.00 | 0.00 | 0.65 | 13 | 0.16 |
|  | Dysphagia | 40 | 0.00 | 0.00 | 0.25 | 39 | 0.31 |
|  | Hearing loss | 47 | -0.00 | 0.00 | 0.60 | 46 | 0.00 |
|  | Visual impairment | 52 | 0.03 | 0.03 | 0.27 | 51 | 0.63 |
| Overall activity | Frailty | 14 | -0.00 | 0.00 | 0.50 | 13 | 0.12 |
|  | Fall | 14 | -0.00 | 0.00 | 0.50 | 13 | 0.53 |
|  | Delirium | 17 | -0.05 | 0.03 | 0.15 | 16 | 0.93 |
|  | Urinary incontinence | 5 | -1.40E-05 | 0.00 | 0.98 | 4 | 0.57 |
|  | Dysphagia | 14 | 0.00 | 0.00 | 0.33 | 13 | 0.29 |
|  | Hearing loss | 14 | -0.00 | 0.00 | 0.51 | 13 | 0.55 |
|  | Visual impairment | 15 | 0.01 | 0.04 | 0.68 | 14 | 0.87 |
| MPA | Frailty | 27 | 0.00 | 0.00 | 0.22 | 26 | 0.52 |
|  | Fall | 27 | 0.06 | 0.03 | 0.06 | 26 | 0.14 |
|  | Delirium | 27 | -0.08 | 0.03 | 0.01 | 26 | 0.52 |
|  | Urinary incontinence | 3 | 0.00 | 0.00 | 0.80 | 2 | 0.89 |
|  | Dysphagia | 11 | -5.65E-05 | 0.00 | 0.89 | 10 | 0.66 |
|  | Hearing loss | 24 | -0.00 | 0.00 | 0.13 | 23 | 0.17 |
|  | Visual impairment | 25 | -0.02 | 0.03 | 0.58 | 24 | 0.91 |
| SB | Frailty | 29 | -0.00 | 0.00 | 0.37 | 28 | 0.16 |
|  | Fall | 31 | 0.02 | 0.02 | 0.41 | 30 | 0.25 |
|  | Delirium | 31 | -0.04 | 0.02 | 0.08 | 30 | 0.12 |
|  | Urinary incontinence | 8 | -0.00 | 0.00 | 0.66 | 7 | 0.29 |
|  | Dysphagia | 19 | -6.69E-05 | 0.00 | 0.85 | 18 | 0.27 |
|  | Hearing loss | 30 | -0.03 | 0.03 | 0.23 | 29 | 0.13 |
|  | Visual impairment | 31 | -0.00 | 0.00 | 0.08 | 30 | 0.33 |
